# Environmental versus geographical effects on genomic variation in wild soybean (*Glycine soja*) across its native range in northeast Asia

**DOI:** 10.1002/ece3.2351

**Published:** 2016-08-14

**Authors:** Larry J. Leamy, Cheng‐Ruei Lee, Qijian Song, Ibro Mujacic, Yan Luo, Charles Y. Chen, Changbao Li, Susanne Kjemtrup, Bao‐Hua Song

**Affiliations:** ^1^ Department of Biological SciencesUniversity of North Carolina at CharlotteCharlotteNorth Carolina28223; ^2^ Gregor Mendel Institute of Molecular Plant BiologyViennaA‐1030Austria; ^3^ Soybean Genomics and Improvement LaboratoryDepartment of AgricultureUSDA‐Agricultural Research ServiceBeltsvilleMaryland20705; ^4^ Department of Bioinformatics and GenomicsUniversity of North Carolina at CharlotteCharlotteNorth Carolina28223; ^5^ Xishuangbanna Tropical Botanical GardenChinese Academy of SciencesYunnan666303China; ^6^ Department of Crop, Soil and Environmental SciencesAuburn UniversityAuburnAlabama36849; ^7^ Biotechnology Assay and Phenotyping GroupMonsanto CompanyDurhamNorth Carolina27709

**Keywords:** Admixture, canonical correlation analysis, environment change, gene flow, natural selection, population genomics

## Abstract

A fundamental goal in evolutionary biology is to understand how various evolutionary factors interact to affect the population structure of diverse species, especially those of ecological and/or agricultural importance such as wild soybean (*Glycine soja*). *G. soja*, from which domesticated soybeans (*Glycine max*) were derived, is widely distributed throughout diverse habitats in East Asia (Russia, Japan, Korea, and China). Here, we utilize over 39,000 single nucleotide polymorphisms genotyped in 99 ecotypes of wild soybean sampled across their native geographic range in northeast Asia, to understand population structure and the relative contribution of environment versus geography to population differentiation in this species. A STRUCTURE analysis identified four genetic groups that largely corresponded to the geographic regions of central China, northern China, Korea, and Japan, with high levels of admixture between genetic groups. A canonical correlation and redundancy analysis showed that environmental factors contributed 23.6% to population differentiation, much more than that for geographic factors (6.6%). Precipitation variables largely explained divergence of the groups along longitudinal axes, whereas temperature variables contributed more to latitudinal divergence. This study provides a foundation for further understanding of the genetic basis of climatic adaptation in this ecologically and agriculturally important species.

## Introduction

**Figure 1:**
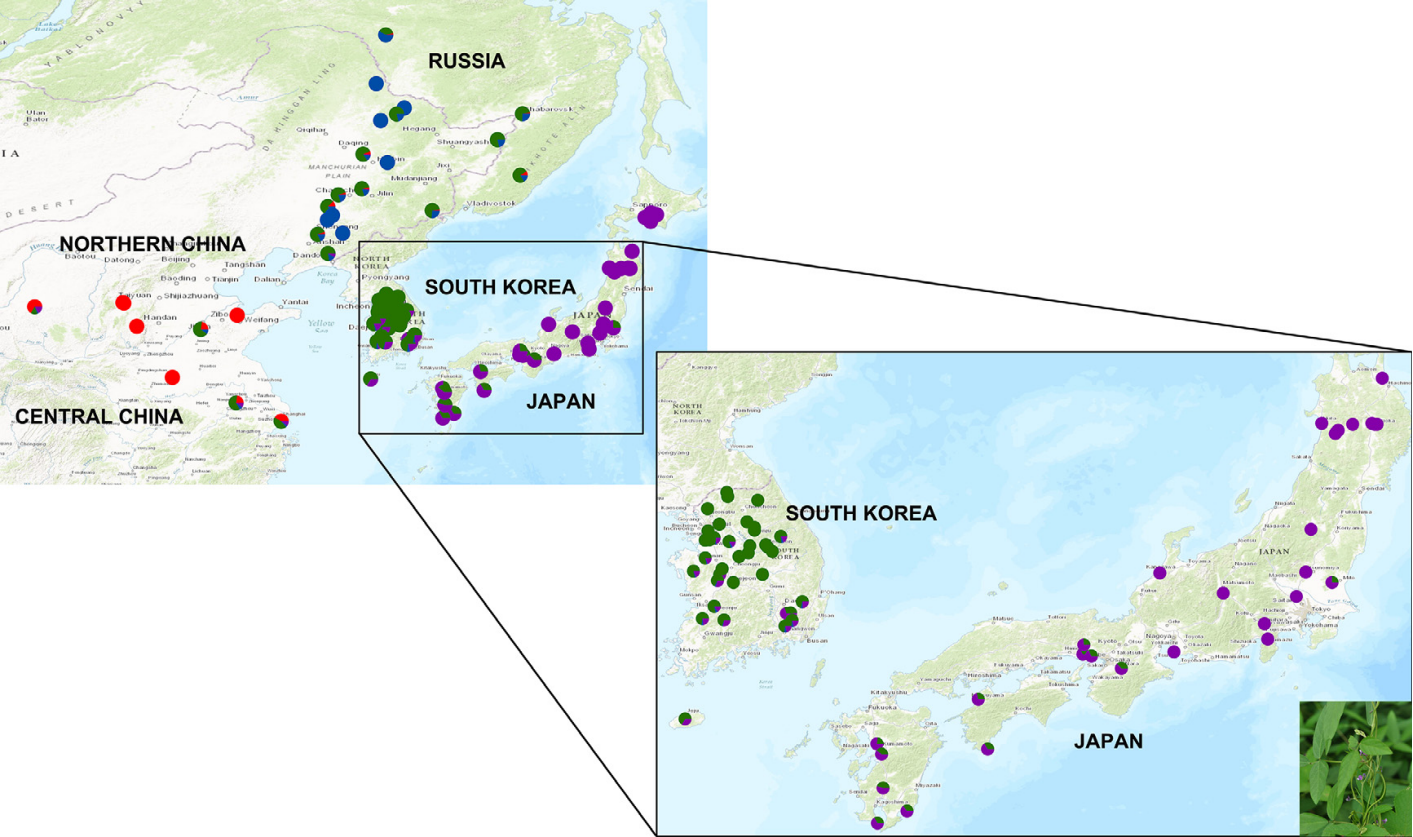
Collection sites of the 99 wild soybean ecotypes sampled across northeastern Asia. The wild soybean ecotypes locations are color‐coded by the genetic cluster to which they were assigned by the STRUCTURE procedure. The four genetic clusters are as follows: Red: GROUP 1; Blue: GROUP 2; Green: GROUP 3; Purple: GROUP 4. Individuals assigned at a probability of <70% were shown on the map with a color Pie indicating the composition of the genome. The plant picture on the top‐left corner is *Glycine soja*.

Contemporary genetic distribution patterns in species are shaped and maintained by their population history and evolutionary factors such as natural selection, gene flow and genetic drift. Understanding the interaction of these factors in natural populations is a long‐standing goal in ecological and evolutionary biology. Currently, there is much interest in assessing the relative contribution of environmental versus geographic factors in shaping population structure, with a number of studies suggesting that environmental adaptation may play an important role in population divergence (Manel et al. 2010; Lee and Mitchell‐Olds 2013; Leamy et al. 2014). Natural selection acts to foster adaptation in local populations and thus also to maintain genetic variation among populations (Mitchell‐Olds and Schmitt 2006). Whether a particular species will adapt to a changing environment, however, depends on the nature and extent of its genetic variation. With current and projected trends in climatic change, it is crucial that we better understand the effects of environmental and geographic factors that shape genetic variation and the adaptation process (Reusch and Wood 2007). This is especially the case for species of both agricultural and ecological significance, such as *Glycine soja* (Fig 1).

The recent availability of genomic data and sophisticated statistical tools has greatly facilitated our understanding of genome‐wide levels of natural variation and population structure in many species (e.g. Lasky et al. 2012; Decker et al. 2014). Increasingly popular are landscape genomics approaches (Manel and Holderegger 2013) that combine genome‐wide variation patterns, large environmental datasets and spatial statistical methods that have been successful in assessing the association between genetic and environmental variables (Fournier‐Level et al. 2011; Hancock et al. 2011; Manel and Holderegger 2013). However, few studies have estimated the relative contribution of environmental and/or other factors to genetic divergence in complex landscapes across a species range. Further, landscape genomics studies typically have been limited to model or well‐studied species for which large amounts of genomic data are available (Sork et al. 2010; Song and Mitchell‐Olds 2011; Ellegren 2014).

East Asia (including the Korea/Tsushima Strait and the East China Sea) harbors the most diverse of the world's temperate flora (review in Harrison et al. 2001; Qiu et al. 2011). This region has experienced dramatic fluctuations in the sea level throughout the Quaternary period (most recent 2.6 million years). This apparently has resulted in population fragmentation during inter/postglacial periods of high sea levels and population admixture during glacial periods with lower sea levels (Qian and Ricklefs 2000). Thus far, only a few genetic studies have been conducted with plant species native to East Asia (Li et al. 2008; Qiu et al. 2009), and much remains to be learned about the different evolutionary factors that maintain species diversity in this region.

The wild soybean, *G. soja*, is native to China, Korea, Japan, and Russia, and there are at least three reasons why it may be considered an ideal species for a landscape genomics study. One reason is its widespread geographic distribution among diverse habitats. Secondly, *G. soja* is the progenitor of the domesticated soybean, *Glycine max*, that is grown throughout many regions of the world and represents one of the most important crops (Mahamed and Ranqappa 1992). The adaptive potential for cultivated soybeans to changing environmental conditions may be limited, however, because the level of genetic variability in *G. max* cultivars has been reduced from that in *G. soja* (Li et al. 2010, 2013, 2014; Song et al. 2013). Given this, it would appear that the gene pool of *G. soja* represents a valuable resource that could be exploited for the improvement of the cultivated soybean, especially if we gain a better understanding of environmental adaptation in this wild species. Finally, large amounts of genomic data have been generated by the soybean research community through genome resequencing and genomic‐wide genotyping and are available to the public (Song et al. 2013; Li et al. 2014; Zhou et al. 2015).

Given these advantages, it is not surprising that this species has been used in increasing numbers of studies. However, most of these studies have focused on the domestication process and/or on traits of agricultural interest, typically with few samples of wild soybeans (Lam et al. 2010; Guo et al. 2012; Li et al. 2014; Zhou et al. 2015). Various other studies have sampled wild soybeans in restricted regions such as Japan or Korea, and have made use of a relatively small number of molecular markers (Kuroda et al. 2006; Kim et al. 2014). Here, we sample wild soybeans across northeast Asia and analyze over 39,000 single nucleotide polymorphisms (SNPs) in each ecotype.

Our study had two basic goals, one of which was to understand the structure and evolutionary processes affecting populations of *G. soja*. Given that *G. soja* primarily is a selfing species (*F*_IS_ = 0.93) (Guo et al. 2012), a reasonable expectation was that our sampled populations would be clearly structured. Some previous studies have suggested genetic clustering in this species (Wen et al. 2009; Li et al. 2010; Kim et al. 2014), but admixture also has been found (Li et al. 2010; Guo et al. 2012). It therefore was of interest to discover the extent of admixture among our sampled wild soybean ecotypes and how it might be understood in terms of the evolutionary history of this species. A second goal was to quantify the contribution of environment versus geography to population differentiation among the wild soybean ecotypes. For this purpose, we made use of 14 different environmental (temperature and precipitation) variables as well as four geographic variables and subjected them to multivariate statistical (canonical correlation and redundancy analysis [RDA]) approaches.

## Materials and Methods

### Data collection and single nucleotide polymorphism genotyping

We selected 99 *G. soja* accessions (interchangeable with ecotypes) from the USDA soybean germplasm collection. Although the USDA has roughly 1035 wild soybean accessions, these represent only 105 unique locations with respect to longitude (LONG) and latitude (LAT), some of which from southern China were not sampled. We focused on northeast Asia and, in this region, sampled one individual at each geographic location (Fig. 1; Table S2) as has been carried out in other studies (Lee and Mitchell‐Olds 2012). We did not attempt to sample additional accessions at each location because some accessions from identical locations with different IDs may have represented the same genotype and could not be distinguished. Further, a structure analysis run with 1035 accessions gave a pattern similar to that for our 99 accession sample (see Results below).

Single nucleotide polymorphism data were extracted from the SoySNP50K dataset (Song et al. 2013). The SoySNP50K dataset consists of 42,509 SNPs for each accession genotyped with the SoySNP50K BeadChip (Song et al. 2013). The SoySNP50K BeadChip, developed at USDA‐ARS, Beltsville, MD, was used to profile 18,484 cultivated soybean accessions and 1168 wild soybean accessions in the USDA Soybean Germplasm Collection and has been widely used for QTL mapping, selection of breeding parents, identification of breeding parents, and GWAS studies (Song et al. 2015). Genotyping with this Beadchip is simple, yields a high throughput with high efficiency, and is cost effective. There are concerns, however, of an ascertainment bias which may result from the fact that SNPs were discovered from a small set of sequenced accessions and then used in arrays for genotyping of larger panels (Ramίrez‐Soriano and Nielsen 2009). As described by Song et al. (2013), the source SNPs for the development of the SoySNP50K Beadchip were identified by comparing the DNA sequence of six cultivated and two wild soybean genotypes. Inclusion of these diverse genotypes as well as SNPs that were evenly selected from euchromatic and heterochromatic chromosomal regions should have greatly reduced the ascertainment bias, although the extent of this bias is difficult to determine.

All SNPs in the dataset had a rate of missing and ambiguous alleles <0.1 (Song et al. 2015). Among the 42,509 SNPs in each accession, 39,616 were polymorphic in our sample and therefore used in the analyses.

### STRUCTURE and phylogenetic analysis

We first used a Bayesian algorithm implemented in the software program STRUCTURE, ver. 2.3.4 (Pritchard et al. 2000) to identify the genetic groups among the wild soybean ecotypes. STRUCTURE was run with different *K* values ranging from 2 to 10, and for each *K* value, five independent runs were performed without using previous population information. Each run consisted of 200,000 cycles of burn‐in followed by 200,000 cycles of model run. To detect the true number of clusters (*K*), we used an ad hoc statistic, Δ*K*, that is based on the rate of change in the log probability of data between successive *K* values (Evanno et al. 2005) (Fig. S1), as well as a consideration of the geographic history of the region (Qiu et al. 2011) and previous studies on this species (Li et al. 2010; Guo et al. 2012).

We found that *K* = 4 was the most plausible value to describe the population structure in our sample (see also the Results below). Using this solution, another longer STRUCTURE run was performed (one million cycles for burn‐in and model run, respectively) that produced Bayesian posterior probabilities that determined the assignment of individual ecotypes to each of the groups. For further analyses, we used all ecotypes with Bayesian posterior probabilities ≥0.7, resulting in a sample of 83. We chose this threshold of 0.7 in part because we wanted to include enough accessions to analyze the considerable admixture revealed by the STRUCTURE procedure (see Results) and because a more stringent threshold would have reduced our sample size even further (with a 0.8 threshold, *N *=* *52). All analyses described below therefore were performed on this sample of 83 ecotypes.

### Genetic distances and PCOA analysis

Using the genetic groups identified by the STRUCTURE procedure, we calculated genetic distances (*F*_ST_ values) between each of the group pairs with the Hierfstat package in R (Goudet 2005). Hierfstat used the variance component method to calculate *F*_ST_ for each SNP, and the results are identical with those calculated with the approach of Weir and Cockerham (1984). Genetic distances for each accession pair also were calculated using a custom R script (publically available at https://gist.github.com/). For each SNP, the three genotypes were coded as 0, 1, and 2, and the genomic distance between each pair of individuals was calculated by averaging the differences across all SNPs with available data for that pair. The genetic distance represented the expected number of different alleles that would be observed in each SNP. To complement the STRUCTURE analysis, we derived a phylogenetic (neighbor‐joining) tree from the pairwise distance matrix using the “nj” function from the package “APE” in R (http://cran.r-project.org/web/packages/ape/index.html).

A principal coordinate analysis (PCOA) of the genetic data was then performed with the pairwise genetic distance matrix, using the PCOA function of the APE package in R (http://cran.r-project.org/web/packages/ape/index.html). PCOA calculated in this fashion is equivalent to a principal components analysis of the allelic states within the wild soybean ecotypes. PCOA was useful in producing a series of axes that could be interpreted as separating two or more of the genetic groups. We plotted the PCOA scores for the first three axes to help visualize their effect in differentiating the groups.

### Environmental and geographic variables

To assess the potential correlation of environmental and geographic variation with the wild soybean genetic structure, it was necessary to use variables that sufficiently captured the general environmental and geographical differences among the northeast Asian regions sampled. For this purpose, we downloaded 19 environmental variables and four geographic variables available at each ecotype site (resolution 30 arc seconds) through WorldClim (Hijmans et al. 2005). The geographic variables included LONG and LAT, both expressed in hundredths of degrees, the maximum change in the elevations between each cell and its eight neighbors (SLOPE), and altitude (ALT), both expressed to the nearest mm. We will refer to these variables collectively as representing “geography,” although they were not meant to exhaustively describe the total geography of a region. The distributions of all these variables were nonnormal, so we transformed them using Box–Cox transformations that generally were successful in promoting normality (Shapiro–Wilk tests: *P *>* *0.05).

From among the 19 environment variables, we chose two temperature and two precipitation variables. The temperature variables (in °C*10, expressed to the nearest tenths) included mean diurnal range (MDR = mean of monthly (maximum temperature – minimum temperature)) and the mean temperature of the wettest quarter (MTW). The precipitation variables (expressed to the nearest mm) included annual precipitation (AP) and precipitation of the wettest month (PWM). These variables tended to show lower correlations with each other and with the geographic variables than other environmental variables. Our intent was to reduce the redundancy inherent among highly associated variables (Hall and Beissinger 2014), and most of our variable pairs showed low to moderate correlations (Table S1).

### Mantel tests

To sort out the potential effects of natural selection (environmental adaptation) versus genetic drift (i.e., isolation by distance) on the genetic variability among the wild soybean accessions, we used Mantel tests of the correlations of genetic, environmental, and geographical distance matrices. We calculated the pairwise genetic distance matrix as described above. With regard to the environmental variables, it was of interest to discover whether nonlinear environmental effects also might impact the genetic structure, as was found by Sork et al. (2010). We therefore derived 10 additional variables from the squares (4) and cross‐products (6) of the original four environment variables and calculated an environmental distance matrix from Euclidean distances between pairs of accessions using all 14 environmental variables. For the geographical distance matrix, we calculated the great‐circle distance (the closest distance between two points on the Earth's surface) in miles from untransformed LONG and LAT values for each pair of accessions using the “fields” package in R (http://CRAN.R-project.org/package=fields).

We conducted Mantel tests of the correlations of both environmental and geographic distances with genetic distances using the VEGAN package in R (http://CRAN.R-project.org/package=vegan). Significance testing of the correlations was performed with 10,000 permutations. We also used partial Mantel tests to discover whether the environmental distances had a significant association with the genetic distances, adjusting for any effects of geographical distances. Significance in this test was interpreted as meaning that genetic variation among the wild soybeans was influenced by environmental selection, whereas a nonsignificant result suggested a role for isolation by distance (genetic drift).

### Canonical correlation analyses

Mantel tests are effective in testing for overall correlations among distance matrices, but suffer from loss of statistical power and are not appropriate for estimating the proportional effect of environmental variables on genetic variation (Legendre and Fortin 2010). We therefore decided to supplement this Mantel approach with another statistical technique from among those available for identifying and estimating the strength of various environmental and geographic factors influencing adaptive genetic diversity in species (review in Schoville et al. 2012). We chose the multivariate technique of canonical correlation analysis (CCA) to examine the relationship of the genetic differences among the wild soybean ecotypes, assessed by the first 15 axes from the PCOA of the genetic SNP data, with the 14 environmental and four geographic variables. This technique is widely used to assess the association of two different sets of variables, even when the variables themselves in each set are correlated (Thompson 1991).

We implemented a CCA with the CANCORR procedure in SAS that generated several canonical axes, each of which consisted of *paired* canonical variables, one genetic canonical variable and one environmental/geographic canonical variable. Each new canonical variable was constructed from linear combinations of the original variables such that the correlation between the pairs was maximized (Legendre and Legendre 1998). CANCORR produced correlations of the original 15 genetic variables with their (genetic) canonical variables and correlations of the original 18 environmental and geographic variables with their canonical variables. The correlations of the environmental and geographic variables with their canonical variables were useful in indicating those that were most associated with the genetic differences among the wild soybean genetic groups.

### Redundancy analysis

We followed up the CCA with a RDA. RDA is a type of ordination that is the multivariate equivalent of linear regression and can be used to test whether the variation in one set of (independent) variables explains the variation in another set of (dependent) variables. If significance is found in this overall test, RDA can then appropriately be used to estimate the extent of the variation that is explained. We used RDA to test whether the 18 environmental/geographic variables could explain variation in the genetic variables (scores from the first 15 axes from the PCOA analysis of the SNP data) and to estimate the percentage contribution of this explained variation to the total variation among the genetic groups. We implemented RDA with the VEGAN package in R (http://CRAN.R-project.org/package=vegan) using a full model that included both genetic and environmental/geographic variables.

To address the independent impact of the environment and geography on genetic differentiation among the wild soybean ecotypes, we ran two partial RDA models. One model included all 14 environmental variables, but adjusted for effects from the four geographic variables (partial environmental model). This model allowed us to test for environmental effects and their contribution to the explained genetic variation that was independent of spatial (geographic) factors. A second model included the four geographic variables, but adjusted for effects from the 14 environmental variables (partial geographic model). This model allowed us to test for geographic effects and their contribution to genetic variation that was independent of the environmental variables. We then summed the estimates of the variation independently explained by environmental and geographic variables and subtracted this sum from the explained variance in the full model to obtain the variance contributed by collinearity of the environment and geography.

## Results

### Genetic groups

**Figure 2:**
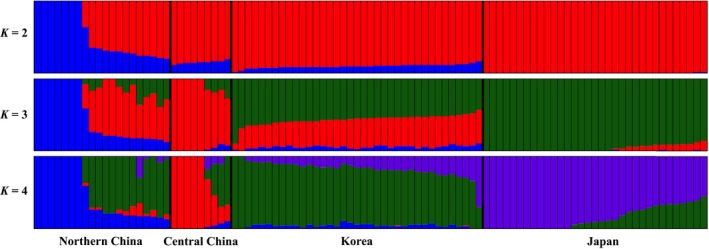
Population structure inferred by the STRUCTURE procedure generated from the 99 wild soybean ecotypes. The colors in each ecotype in A for *K* = 2, 3, and 4 represent the fraction of their genome that is inferred to be from each of four genetic groups. Red: GROUP 1; Blue: GROUP 2; Green: GROUP 3; Purple: GROUP 4.

The STRUCTURE procedure applied to the wild soybean SNP data identified four major genetic groups (GROUP 1‐GROUP 4). These groups primarily describe ecotypes from separate geographical regions: GROUP 1 – central China (*N *=* *5); GROUP 2 – northern China (*N *=* *7); GROUP 3 – Korea (*N *=* *45); and GROUP 4 – Japan (*N *=* *26) (Fig. 1). When *K* = 2, the samples from northern China were separated from all others; when *K* = 3, samples from northern China were further separated from all others and from those in central China; when *K* = 4, four separate groups were defined (Fig. 2). The membership of each individual generated in this procedure is listed in Table S2. The phylogenetic tree generated from the SNP data (Fig. S2) also clearly separates the four groups. The means of the eight (untransformed) geographic and environmental variables in each of the four genetic groups are given in Table S3.

The *F*_ST_ values calculated between each of the four groups are given in Table 1. These values range from 0.098 to 0.655, averaging 0.44. The highest value is between GROUP 1 and 2 and the lowest between GROUP 3 and 4. *F*_ST_ values for GROUP 1 or 2 versus GROUP 3 or 4 are intermediate in magnitude. In general, these values show that GROUP 3 and 4 are much more closely related than is either of these groups to GROUP 1 or to GROUP 2.

**Table 1:** *F*_ST_ values between each pair of the genetic groups

	Group1	Group2	Group3
Group2	0.655		
Group3	0.321	0.381	
Group4	0.256	0.287	0.098

### Principal coordinates analysis

The first 15 axes from the PCOA of the genetic data (Table 2) explain nearly 49% of the total variance in our wild soybean sample. The first coordinate axis (PCOA1) explains over 13% of the variation, whereas the second and third axes explain only about one‐half of this amount. All remaining axes explained <4% of the variation.

**Table 2:** Principal coordinate analysis results

PCOA	Eigenvalue	Proportion	Cumulative
PCOA1	0.506	0.135	0.135
PCOA2	0.258	0.069	0.204
PCOA3	0.229	0.061	0.265
PCOA4	0.115	0.031	0.296
PCOA5	0.094	0.025	0.321
PCOA6	0.080	0.021	0.342
PCOA7	0.075	0.020	0.362
PCOA8	0.065	0.017	0.379
PCOA9	0.062	0.017	0.396
PCOA10	0.062	0.017	0.412
PCOA11	0.059	0.016	0.428
PCOA12	0.057	0.015	0.443
PCOA13	0.053	0.014	0.458
PCOA14	0.053	0.014	0.472
PCOA15	0.051	0.014	0.485

Shown are the eigenvalues and the proportion of the variation they explain for the first 15 axes derived from principal coordinate analysis of the genetic data from wild soybeans.

**Figure 3:**
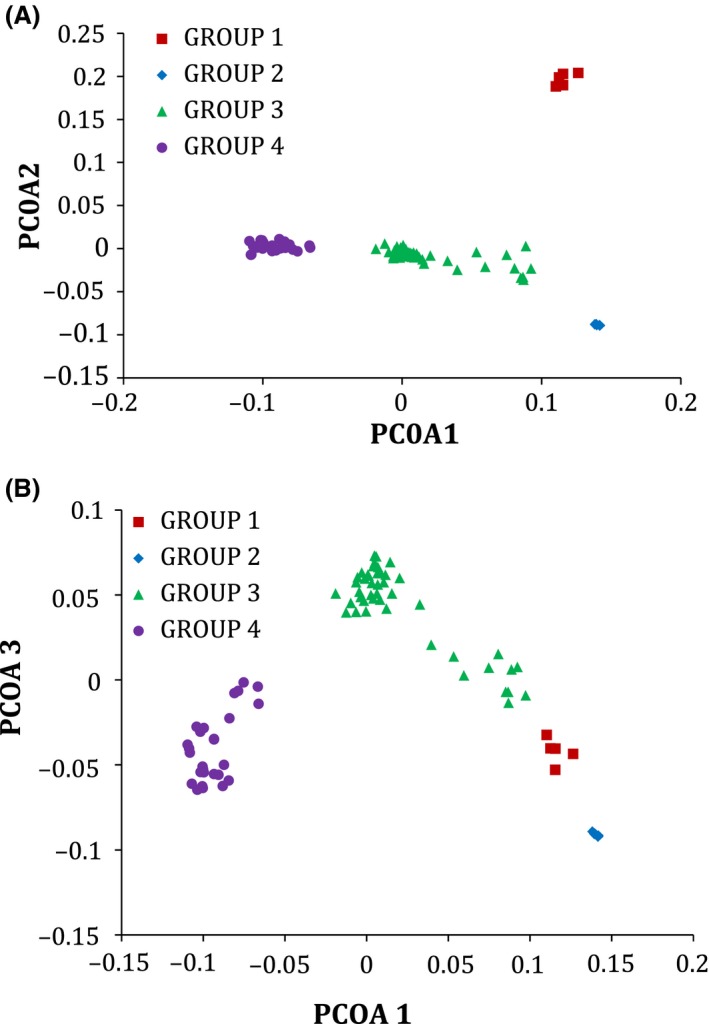
Plots of the scores from the first (PCOA1) and second (PCOA2) axis (A) and the first and third (PCOA3) axis (B) derived from a principal coordinates analysis of the genetic (SNP) data from 83 wild soybean ecotypes.

Figure 3 shows plots of the scores from the first three PCOA axes generated from the genetic data for the wild soybean sample. The plot of the first versus the second axis clearly shows four discrete clusters corresponding to the four groups identified in the STRUCTURE procedure. PCOA1 distinguishes among GROUP 4, GROUP 3, and the two China groups (GROUP 1 and GROUP 2). PCOA2 separates GROUP 1 (central China) from the other groups and PCOA3 is helpful in distinguishing GROUP 2 (northern China) from the other groups.

### Genetic and environmental/geographical associations

In the Mantel tests, correlations of the genetic distances with both the environmental (*r *=* *0.59) and the geographic distances (*r *=* *0.75) were high and statistically significant (*P *<* *0.0001). In the partial Mantel test, the genetic and environmental distances still were significantly correlated (*r *=* *0.094, *P *=* *0.029) after accounting for geographic distance. This result suggests that, after controlling for isolation by distance alone, environmental selection may well have played a role in shaping genetic variability among the wild soybean accessions. Specific effects of the environmental variables are examined in the CCA results below.

The first seven pairs of canonical variables produced from the CCA of the 15 genetic variables (PCOAs) with the 18 environmental/geographical variables in the wild soybean sample were statistically significant (Table 3). The canonical correlations associated with these variables range from 0.687 to nearly 1.0 (0.983), indicating a high degree of association between the genetic and the environmental/geographical variables. Cumulatively, these seven pairs account for a very high percentage (97.1%) of the explained variation, with the first three pairs alone contributing 85.4%.

**Table 3:** CCA of the genetic and environmental/geographic variables in the wild soybean sample

Canonical axes	Canonical correlation	% Explained	*F* value	Prob > *F*
I	0.983	47.7	5.27	<0.0001
II	0.961	19.6	3.97	<0.0001
III	0.958	18.2	3.12	<0.0001
IV	0.877	5.4	2.23	<0.0001
V	0.811	3.2	1.79	<0.0001
VI	0.711	1.7	1.48	0.0018
VII	0.687	1.5	1.31	0.0330

CCA, canonical correlation analysis.

Shown are the canonical correlations, percentage explained, the *F* value, and the probability of the *F* values for all statistically significant canonical variables. These values were derived from CCA of the first 15 axes from PCOA of the wild soybean genetic data with 18 environmental and geographical variables.

Correlations of the first three pairs of genetic (PCOA) and environmental/geographical variables with their canonical variables are shown in Table 4. The first genetic canonical variable is highly correlated with PCOA1, the first principal coordinate. The first environmental/geographical canonical variable is most highly correlated with LONG. It also is correlated with mean diurnal temperature range (MDR) and especially AP, their squares and their cross‐product as well as an interaction of AP with precipitation in the wettest month (PWM). Thus, this first environment/geographical canonical variable consists of a complex effect involving LONG and both linear and nonlinear AP and diurnal temperature range variables and interactions with both precipitation variables. Because this canonical variable is highly associated with PCOA1, this complex of environmental and geographic variables is involved in the differentiation of GROUP 4 from GROUP 3 and the two China groups (GROUP 1 and 2).

**Table 4:** Correlations of genetic (PCOA scores) and environmental/geographical variables with their canonical variables

	I	II	III
Genetic			
PCOA1	0.948	−0.080	0.167
PCOA2	0.240	0.223	−0.876
PCOA3	0.095	0.814	0.289
PCOA4	−0.086	0.477	−0.075
PCOA5	0.111	−0.106	0.123
PCOA6	−0.035	0.022	0.088
PCOA7	−0.036	−0.064	0.084
PCOA8	0.066	0.016	0.134
PCOA9	−0.028	0.121	0.137
PCOA10	0.013	0.066	0.181
PCOA11	−0.031	0.123	−0.066
PCOA12	0.028	−0.013	−0.078
PCOA13	−0.009	0.056	0.007
PCOA14	0.070	0.024	0.064
PCOA15	0.031	0.003	−0.029
Environmental/Geographical			
LONG	**−0.867**	−0.406	0.102
LAT	0.378	**−0.770**	0.412
SLOPE	−0.107	0.207	−0.007
ALT	0.214	−0.130	−0.356
MDR	**0.747**	0.154	0.165
MTW	−0.020	**0.547**	−0.115
AP	**−0.842**	0.334	−0.040
PWM	−0.208	**0.841**	0.319
MDR^2^	**0.750**	0.116	0.141
MTW^2^	−0.002	0.489	−0.212
AP^2^	**−0.770**	0.223	−0.112
PWM^2^	−0.196	**0.844**	0.319
MDR*MTW	0.211	**0.591**	−0.064
MDR*AP	**−0.706**	**0.587**	−0.128
MDR*PWM	0.593	0.491	0.298
MTW*AP	−0.474	**0.582**	−0.015
MTW*PWM	−0.036	**0.632**	−0.051
AP*PWM	**−0.778**	0.441	0.009

MDR, mean diurnal temperature range; MTW, mean temperature of the wettest quarter; AP, annual precipitation; PWM, precipitation of wettest month; LONG, longitude; LAT, latitude; SLOPE, the maximum change in the elevations between each cell and its eight neighbors; ALT, altitude in meters.

Shown are correlations of the 15 genetic variables (PCOA1‐PCOA15) with the scores from their first three canonical variables, and correlations of 18 environmental/geographical variables with the scores from their first three canonical variables. Several of the highest correlations of the environmental/geographical variables with their canonical variables are shown in bold for emphasis.

The second genetic canonical variable loads most highly on PCOA3, the principal component previously seen to separate GROUP 2 from the other groups. The second environmental/geographic variable correlates highly with LAT, PWM, and PWM^2^. There are also moderately high correlations of this canonical variable with mean temperature in the wettest quarter (MTW) and interactions of both temperature variables (MDR and MTW) with each other and with AP. This suggests that besides LAT differences, precipitation (especially in the wettest month), mean temperature of the wettest quarter (MTW), and several temperature‐related interactions are the factors that distinguish wild soybean ecotypes from central China from those in other groups.

The third genetic canonical variable loads most heavily on PCOA2 that previously was seen to separate GROUP 1 ecotypes from the others. Most of the environmental and geographical variables show low correlations with this canonical variable, although there is some association with LAT and ALT as well as with precipitation in the wettest month (PWM) and its square. Thus, there is evidence that beyond LAT and slope differences, precipitation in the wettest month accounts for some of the differentiation of the northern China ecotypes from all others.

### Partitioning of variance

The RDA showed that the full model using all 18 environmental and geographic variables was highly significant (*P *<* *0.001), with this model explaining 64.0% of the total genetic variation among the wild soybean ecotypes. The partial environment model also showed significance (*P *<* *0.001), suggesting that the environmental variables account for some of the genetic differentiation in the wild soybean ecotypes; that is, populations in the different geographic regions have adapted to different environments. In the partition of the total genetic variation (Fig. S3), environmental effects independently contributed nearly 24%, much more than geographic effects (6.6%). Joint (collinear) effects of both of these factors contributed nearly 34%, with the remaining variance (36%) being unexplained.

## Discussion

### Genetic structure and admixture in wild soybeans

Using a large number of SNPs genotyped in the wild soybean ecotypes, our population structure analysis resulted in four genetic groups largely corresponding to four separate geographic regions (central China, northern China, Korea, and Japan, Figs 1 and 2). Li et al. (2010) found separate genetic clusters for *Glycine soja* in Japan and Korea as we did, as well as three additional clusters in China corresponding to northern, central (Huang‐Huai Valley), and southern regions. Our study focused on northeast Asia (Fig. 1), and thus we did not include samples from southern China. With additional accessions from this southern China region, we may well have found three China subclusters as well.

The *F*_ST_ values we calculated between the pairs of groups clearly showed that the greatest genetic difference was between GROUP 2 (northern China) and all other groups, especially GROUP 1 (central China, *F*_ST_ = 0.655, Table 1). Guo et al. (2012) also found high levels of genetic (microsatellite) differentiation among 39 wild soybean populations in China, with their *F*_ST_ estimates averaging 0.56. Across the central and northern regions in China, therefore, these results suggest that there has been limited gene flow. In contrast, the lowest *F*_ST_ value (0.098) we found was between GROUP 3 and 4, with ecotypes in these groups found in Korea and Japan.

While the wild soybean ecotypes clearly showed a population structure, they also exhibited a surprisingly high level of admixture (Figs 1 and 2), and there are several potential factors that explain this. An important possibility is intensive gene flow between populations, especially between ecotypes in GROUP 3 and 4 that are found primarily in the geographically proximate regions of South Korea and Japan. Presently, these countries are isolated by the Korea/Tsushima Strait, a barrier that may appear formidable for a largely inbreeding species with a low estimated outcrossing rate of 3.4% (Kuroda et al. 2006), but the past Japan/Korea land connection should have fostered gene flow between these regions. Further, a spatial autocorrelation analysis of Japanese populations revealed a close population genetic relationship for wild soybeans within a radius of 100 km (Kuroda et al. 2006), whereas the shortest distance between South Korea and Japan is only about 50 km. The *F*_ST_ value between GROUP 3 and 4 of 0.1 corresponds to a number of migrants (*N*_m_) equal to 9 (Slatkin and Barton 1989), also implying that there has been abundant gene flow across these two regions.

GROUP 1 and 2 also showed some admixture, and it is possible that gene flow might explain this as well. In particular, some of the admixture in GROUP 2 (northern China) ecotypes may have resulted from a unidirectional movement (Korea to northern China) in this species due to climate changes. It also is likely that the admixture in both Chinese groups can be explained by their small sample sizes.

Incomplete lineage sorting previously has been suggested as a possible mechanism that explains why wild soybeans from southern China are more related to those from northern rather than central China (Guo et al. 2012). For the wild soybean ecotypes we sampled, we cannot exclude lineage sorting as a possible explanation for the admixture we found. The greatest level of admixture was seen for ecotypes in GROUP 3 and 4, however, and incomplete lineage sorting would be expected to generate a more even admixture distribution, not the gradient actually seen (Fig. 1).

The dramatic fluctuations in the sea level and climate in East Asia throughout the past 1.5 million years (Qiu et al. 2011), together with various evolutionary factors acting on these populations, may explain the diversity patterns of the wild soybean ecotypes we sampled, particularly in Korea and Japan. During the ice age, many plants presumably died because of the freezing temperatures, although some may have survived in refugia thought to have existed in Korea and Japan (Qiu et al. 2011). During these periods, populations of wild soybeans may have been small enough in size such that genetic drift was an important factor influencing their level of genetic variability. As expansion of these populations occurred, it is reasonable to assume that because of environmental heterogeneity, selection would have been an important factor influencing their genetic structure. At the same time, lowered sea levels would have linked ecotypes in Japan and Korea via a land bridge, thus permitting extensive gene flow that would account for the admixed wild soybean ecotypes we found especially in Korea and Japan.

### Environmental heterogeneity influences wild soybean population differentiation

The CCA demonstrated a strong association of the environmental/geographical and genetic variables across the range of wild soybeans we sampled. The first genetic canonical variable was highly associated with PCOA1 that separated wild soybean ecotypes from the continent, the Korean peninsula, and the Japanese islands (see Fig. 3). The four genetic groups differed more in LONG (ANOVA *F *=* *159.2, *P *<* *0.0001) than in LAT (ANOVA *F *=* *10.8, *P *<* *0.0001), so it was not surprising to see that the first environmental/geographical canonical variable was highly correlated with LONG. In our sample, LONG reflects proximity to the ocean, a factor that especially influences precipitation in this region. Thus, mean values for AP for ecotypes in GROUP 3 and 4 that are closer to the ocean are higher (1129 and 1504; Table S3) than those for GROUP 1 and 2 (157 and 172). As a consequence, AP was the most important contributor to the first environmental/geographical canonical variable that was responsible for the separation of these two groups from the other two continental (China) groups.

The interaction of the two precipitation variables, AP and precipitation in the wettest month (PWM), also contributed heavily to the first environmental/geographic canonical variable (Table 4). This suggests that the importance of PWM depends on the level of AP. Regression plots (Fig. S4‐A) show that in areas experiencing mean or high levels of AP, increases in PWM increase PCOA1 scores that differentiate genetic differences among the accessions. In areas of low AP levels, increases in PWM tend to slightly decrease genetic differences (i.e., PWM is not as important in differentiating the groups). The interaction of AP and MDR also contributed to the first environmental/geographic canonical variable and was produced primarily by a trend of increase in genetic differentiation with increasing MDR that is greatest in areas of low AP levels (Fig. S4‐B).

The second genetic canonical variable was most highly correlated with PCOA3 that separated GROUP 2 (northern China) from the other groups (Fig. 3). The most important environmental variable associated with the second environmental/geographic canonical variable was precipitation in the wettest month, PWM. Even though the mean PWM value for GROUP 2 is intermediate compared to those in the other groups, its importance probably is ascribable once again to the high mean PWM value for GROUP 3 that was differentiated to some extent by PCOA3 (Fig. 3). The second environmental/geographic canonical variable also was associated with LAT differences among the groups (primarily GROUP 2 vs. the others), where temperature effects would have been expected to make a greater contribution. In our soybean sample, LAT was moderately correlated with mean temperature of the wettest month, MTW (*r *= −0.64, *P *<* *0.0003), although not with mean diurnal temperature range, MDR (*r *=* *0.25, *P *=* *0.0502). MTW was most highly associated with the second environmental/geographical canonical variable (correlation of 0.547, see Table 4) compared to first and third canonical variables. Both temperature variables, MDR and MTW, also contributed to the second environmental/geographical canonical variable through interactions with all three other environmental variables, so temperature effects were important in differentiating GROUP 2 (northern China) from the others.

The CCA showed that populations in different geographical regions experience different environments, with precipitation especially associated with longitudinal population differences and temperature with latitudinal population differences. Overall, the variance partitioning procedure estimated that the environmental contribution to the genetic differentiation among the wild soybean ecotypes was 23.6%. Interactions of temperature and precipitation were an important component of the total environmental impact, as a preliminary variance partition performed without these interactions reduced the independent environmental contribution to 16.1%. This suggests that in addition to individual temperature or precipitation effects themselves, particular combinations of temperature and precipitation are important in population differentiation and adaptation in the wild soybean ecotypes. Accumulating numbers of studies have demonstrated a role for various precipitation and/or temperature environmental variables in shaping genetic differences among populations of various species (Nakazato et al. 2008; Pease et al. 2009; Manel et al. 2010; Keller et al. 2011; Vincent et al. 2013; Abebe et al. 2015), but very few have used nonlinear environmental variables in these analyses. An exception is a study by Sork et al. (2010) who conducted a CCA of genetic differentiation in California valley oaks and found that the contribution of the first four canonical axes averaged 16.7% for linear environmental effects but 27.3% for nonlinear environmental components. That study and our results therefore suggest that environmental effects on population differentiation may be more complex than can be captured in linear models and that their interactions are well worth considering for inclusion.

Our analysis showed that environmental effects (23.6%) contributed much more than geographic effects (6.6%) to the differentiation of the wild soybean genetic groups. There was considerable collinearity (33.8%) between both of these factors, probably because of the wide geographic sampling and some moderate correlations of the precipitation and temperature variables with LONG and LAT. As far is known, our study is the first to partition the genetic variance among wild soybeans into environmental and geographic components, and therefore no comparable studies are available for comparison. Environmental and geographic factors have been assessed in other species and, as expected, their contributions vary. For example, Sork et al. (2010) found that the independent environmental and geographic contributions, respectively, to the explained genetic differentiation in California valley oaks were 34% and 14% for the first canonical axis. In a landscape genomics study of local adaptation in barley landraces from 61 sites in Ethiopia, Abebe et al. (2015) discovered that environmental (primarily temperature and precipitation) differences contributed 40%, and geographic effects 29%, of the explained genetic variation. Lee and Mitchell‐Olds (2011) estimated that environmental, geographic, and environmental/geographic joint effects contributed 21.6%, 0.05%, and 4.80%, respectively, to the total genetic divergence between two major genetic groups (east and west) of *Boechera stricta*. More studies are needed to gain an understanding of the general pattern of environmental and geographic contributions and especially how the individual variables comprising these factors act to differentiate genetic groups in various populations.

## Conclusions and Perspective

Wild soybeans provide a unique and interesting study system with both ecological and agricultural significance. We used a large genomic dataset in this species to understand its population structure and the contribution of both environment and geography to population differentiation. We discovered that wild soybean populations showed a clear genetic structure with high levels of admixture that possibly are due to gene flow among populations. This provides a basis for the next phase of our study that will focus on identifying for quantitative trait loci affecting environmental adaptation in wild soybeans, as has been successfully carried out in other plant species with extensive population genomic data, such as *Arabidopsis* (Hancock et al. 2011), *Medicago* (Yoder et al. 2014), and Loblolly pine (Eckert et al. 2010).

## Data Accessibility

The data can be accessed in the Soybase database (http://soybase.org/) by downloading the specific accessions we used.

## Conflict of Interest

None declared.

## Supporting information


**Table S1**. Correlations among the geographical and environmental variables for soybean the soybean sample.
**Table S2.** Membership for the 99 soybean accessions in each of the four groups identified by the STRUCTURE procedure.
**Table S3.** Means of the eight (untransformed) geographic and environmental variables for each of the four genetic groups.
**Figure S1.** Statistics from the STRUCTURE analysis (Evanno et al. 2005) of the wild soybean genetic data suggest that the most plausible number of genetic groups (*K* value) is 4.
**Figure S2.** Neighbor‐joining tree generated from the 99 wild soybean ecotypes.
**Figure S3.** A venn diagram showing the proportion of genomic variance explained by environmental variables (light blue), geography (light green), and the joint effects of environment and geography (blue‐green).
**Figure S4.** Linear regression plots illustrating the joint effects of PWM on PCOA1 (A) and MDR on PCOA1 (B) at the mean of AP and at 2 standard deviations above and below the AP mean.
