# Supplementary material for: Environmental versus geographical effects on genomic variation in wild soybean (Glycine soja) across its native range in northeast Asia
Source: Ecol Evol. 2016 Aug 14;6(17):6332–44. doi: 10.1002/ece3.2351 (PMC5016653; doi:10.1002/ece3.2351)

Appendices

Table S1. Correlations among the geographical and environmental variables for soybean the soybean sample.

	MDR	MTW	AP	PWM	LONG	LAT	SLOPE
MTW	-0.03						
AP	-0.68**	0.13					
PWM	-0.03	0.42**	0.55**				
LONG	-0.76**	-0.28**	0.58**	-0.15			
LAT	0.25*	-0.64**	-0.59**	-0.59**	0.07		
SLOPE	-0.01	-0.23	0.18	0.17	0.08	-0.10	
ALT	0.36**	-0.52**	-0.23	-0.29*	-0.17	0.17	0.54**

MDR = mean diurnal temperature range, MTW = mean temperature of the wettest quarter, AP = annual precipitation, PWM = precipitation of wettest month. LONG = longitude, LAT = latitude, SLOPE = the maximum change in the elevations between each cell and its eight neighbors, and ALT = altitude in meters.
* = $P < 0.05$; ** = $P < 0.01$ from the false discovery rate procedure.

Table S2. Membership for the 99 soybean accessions in each of the four groups identified by the STRUCTURE procedure. Longitude and latitude values that give the origin for each accession also are listed.

Samples	PI #	Long	Lat	Group1	Group2	Group3	Group4
s01	PI101404B	126.07	46.2	0.065	0.125	0.78	0.031
s02	PI339732	128.54	37.31	0	0.053	0.767	0.18
s03	PI339871A	126.5	33.42	0	0	0.663	0.337
s04	PI366120	140.38	39.53	0	0	0	1
s05	PI366122	139.84	37.46	0	0	0	1
s06	PI366123	141.25	39.7	0	0	0	1
s07	PI366125	139.53	36.03	0.001	0.001	0.117	0.881
s08	PI378683	136.62	36.53	0	0	0.18	0.82
s09	PI378686A	132.95	32.78	0	0	0.337	0.663
s10	PI378691	131.23	31.46	0	0	0.352	0.648
s11	PI378698	138.85	35.45	0	0	0.095	0.905
s12	PI406684	142.44	42.87	0	0	0	1
s13	PI407036	140.36	39.5	0	0	0	1
s14	PI407037	140.73	39.7	0	0	0	1
s15	PI407044	140.42	39.57	0	0	0	1
s16	PI407047	140.07	39.72	0	0	0	1
s17	PI407050	141.14	39.72	0	0	0	1
s18	PI407053	137.97	36.1	0	0	0.105	0.895
s19	PI407056	136.92	34.85	0	0	0.066	0.934
s20	PI407089	134.98	34.8	0	0	0.338	0.662
s21	PI407097	135.16	34.76	0	0	0.322	0.678
s22	PI407120	135	35	0	0	0.263	0.737
s23	PI407144	130.59	32.89	0	0	0.234	0.766
s25	PI407167	127.12	37.28	0	0.033	0.777	0.19
s26	PI407174	127.44	37.2	0	0.059	0.789	0.152
s27	PI407184	127.02	37.24	0	0.03	0.812	0.158
s28	PI407198	127.82	37.62	0	0.08	0.8	0.12
s29	PI407201	127.98	37.44	0	0.047	0.841	0.111
s30	PI407202	127.98	37.5	0	0.052	0.812	0.135
s31	PI407209	127.88	37.11	0	0.028	0.868	0.104
s32	PI407217	127.85	36.96	0	0.043	0.846	0.11
s33	PI407221	127.65	36.88	0	0.047	0.877	0.075
s34	PI407229	127.24	36.5	0	0.027	0.77	0.203
s35	PI407235	127.29	36.62	0	0.013	0.844	0.143
s36	PI407240	128.75	35.6	0	0.018	0.718	0.264
s37	PI407246	128.75	35.68	0	0.03	0.721	0.249

s38	PI407249	128.66	35.67	0	0.001	0.297	0.702
s39	PI407254	128.78	35.52	0	0.053	0.735	0.213
s40	PI407267	128.63	35.4	0	0.065	0.727	0.208
s41	PI407271	126.87	35.56	0.001	0.048	0.727	0.224
s42	PI407275	126.99	37.43	0	0.048	0.882	0.07
s43	PI407278	127.23	37.57	0	0.035	0.866	0.099
s44	PI407298	123.48	41.64	0.055	0.184	0.761	0
s45	PI407302	118.85	32.06	0.23	0.074	0.599	0.097
s46	PI407304	121.41	31.02	0.417	0.042	0.442	0.099
s47	PI407322	127.53	36.33	0	0.037	0.815	0.148
s48	PI424032	126.98	37.9	0	0.037	0.862	0.1
s49	PI424059B	127.39	38.25	0	0.019	0.917	0.064
s50	PI424063	127.41	38.16	0	0	1	0
s51	PI424064	128.05	38.08	0	0	1	0
s52	PI424088	128.22	37.13	0	0.041	0.783	0.176
s53	PI424091A	128.26	37.08	0	0.056	0.871	0.073
s54	PI424093	128.36	36.99	0	0.096	0.807	0.097
s55	PI424096	127.19	36.37	0.01	0.028	0.672	0.29
s56	PI424102A	128.15	36.5	0	0.02	0.836	0.143
s57	PI424117	129	35.92	0	0.048	0.713	0.24
s58	PI458537A	127.45	45.73	0	1	0	0
s59	PI458540D	133.71	47.02	0	0.213	0.787	0
s60	PI464866A	127.07	48.11	0	1	0	0
s61	PI464889C	124.66	43.89	0.052	0.206	0.742	0
s62	PI464891B	126	44.23	0.033	0.167	0.772	0.028
s63	PI464925C	124.06	43.22	0.127	0.176	0.697	0
s64	PI464926	124.33	42.72	0	1	0	0
s65	PI464927A	124.04	42.46	0	1	0	0
s66	PI464928	124.91	41.71	0	1	0	0
s67	PI468396B	113.2	36.42	1	0	0	0
s68	PI468397A	112.47	37.73	1	0	0	0
s69	PI468398B	112.42	37.76	1	0	0	0
s70	PI468399B	118.91	37.04	1	0	0	0
s71	PI483466	116.84	36.24	0.227	0.095	0.678	0
s72	PI483468A	115.23	33.51	1	0	0	0
s73	PI486220	138.92	35.12	0	0	0.091	0.909
s74	PI487428	141.2	39.7	0	0	0.047	0.953
s75	PI487430	142.1	42.6	0	0	0	1
s76	PI487431	130.6	31.2	0	0	0.331	0.669
s77	PI507582	141.36	40.68	0	0.001	0	0.999
s78	PI507601B	140.29	36.33	0	0	0.25	0.75

s79	PI507609	139.73	36.55	0	0	0.075	0.925
s80	PI507632	135.8	34.5	0	0	0.372	0.628
s81	PI507644	132.75	33.84	0	0	0.288	0.712
s82	PI507657	130.72	31.95	0	0	0.451	0.549
s83	PI507667	130.69	32.67	0	0	0.391	0.608
s84	PI507757	144.03	64.41	0.177	0.174	0.355	0.294
s85	PI507805	135	45	0.094	0.161	0.744	0
s86	PI508067	142.41	42.37	0	0	0	1
s87	PI514674	142.75	42.76	0	0.002	0	0.998
s88	PI522179	126.82	50.21	0	1	0	0
s89	PI522180	128.41	48.82	0	1	0	0
s90	PI522182A	127.97	48.48	0	0.21	0.789	0
s91	PI532453A	130	43	0.026	0.263	0.711	0
s92	PI549037	124.07	40.55	0	0.146	0.776	0.077
s93	PI549046	107.4	37.53	0.663	0.024	0.146	0.168
s94	PI562531	126.93	37.23	0	0.047	0.799	0.154
s95	PI562544	126.93	36.85	0.009	0.026	0.776	0.188
s96	PI562550	126.68	36.57	0	0.027	0.784	0.189
s97	PI562556	127.12	35.82	0	0.062	0.785	0.153
s98	PI562565	127.33	35.53	0	0.022	0.701	0.277
s99	PI578336	127.36	52.98	0.041	0.586	0.372	0
s100	PI578345	135.13	48.5	0.032	0.258	0.71	0

Table S3. Means of the eight (untransformed) geographic and environmental variables for each of the four genetic groups. MDR = mean diurnal temperature range, MTW = mean temperature of the wettest quarter, AP = annual precipitation, PWM = precipitation of wettest month. LONG = longitude, LAT = latitude, SLOPE = the maximum change in the elevations between each cell and its eight neighbors, and ALT = altitude in meters. Means for MDR and MTW are expressed in centigrade degrees X 10 whereas those for AP and PWM are expressed in mm.

	Group I<br>Central China	Group II<br>Northern China	Group III<br>Korea	Group IV<br>Japan
LONG	114.4	126.1	127.8	138.9
LAT	36.5	45.7	38.8	38.1
SLOPE	152.8	54.4	172.6	161.2
ALT	618.4	279.6	163.1	200/0
MDR	123.0	126.3	110.6	89.5
MTW	227.4	201.6	224.7	209.2
AP	577.2	661.3	1128.9	1503.6
PWM	156.8	171.9	28.2	203.2

Figure S1. Statistics from the STRUCTURE analysis (Evanno *et al*. 2005) of the wild soybean genetic data suggest that the most plausible number of genetic groups (K value) is 4.

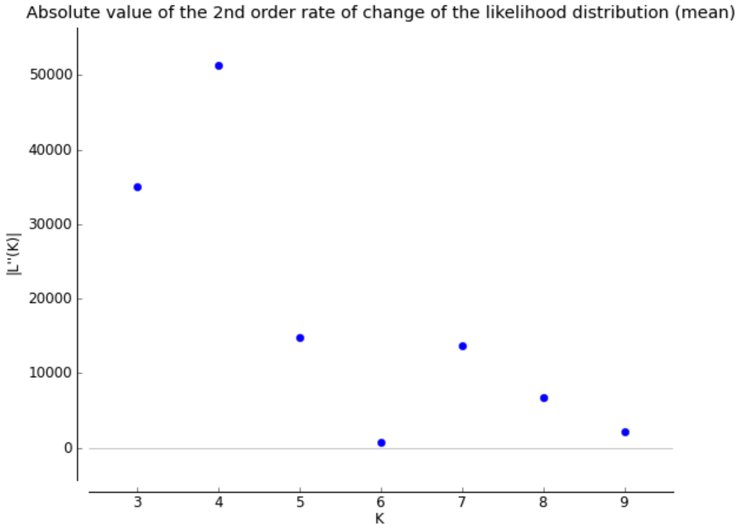

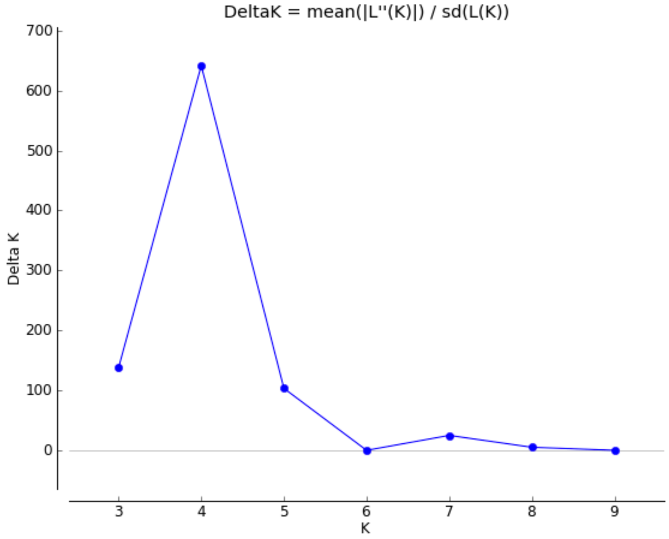

Figure S2. Neighbor-joining tree generated from the 99 wild soybean ecotypes. Red: GROUP 1; Blue: GROUP 2; Green: GROUP 3; Purple: GROUP 4.

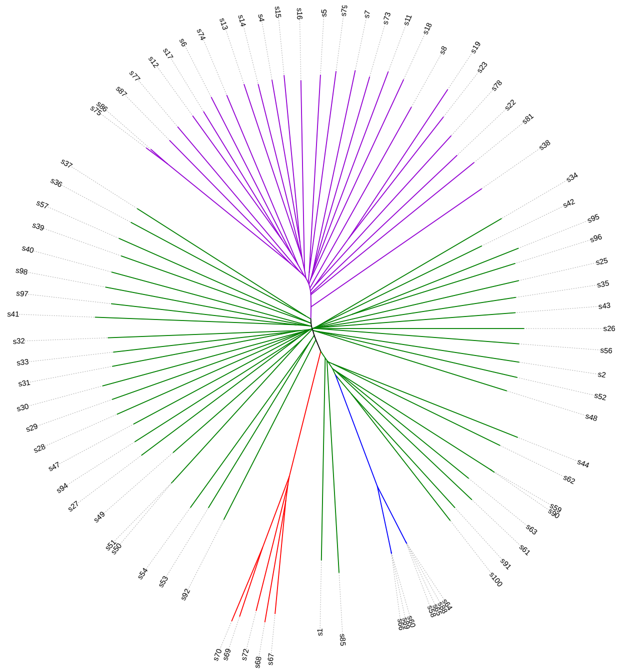

Figure S3. A venn diagram showing the proportion of genomic variance explained by environmental variables (light blue), geography (light green), and the joint effects of environment and geography (blue-green).

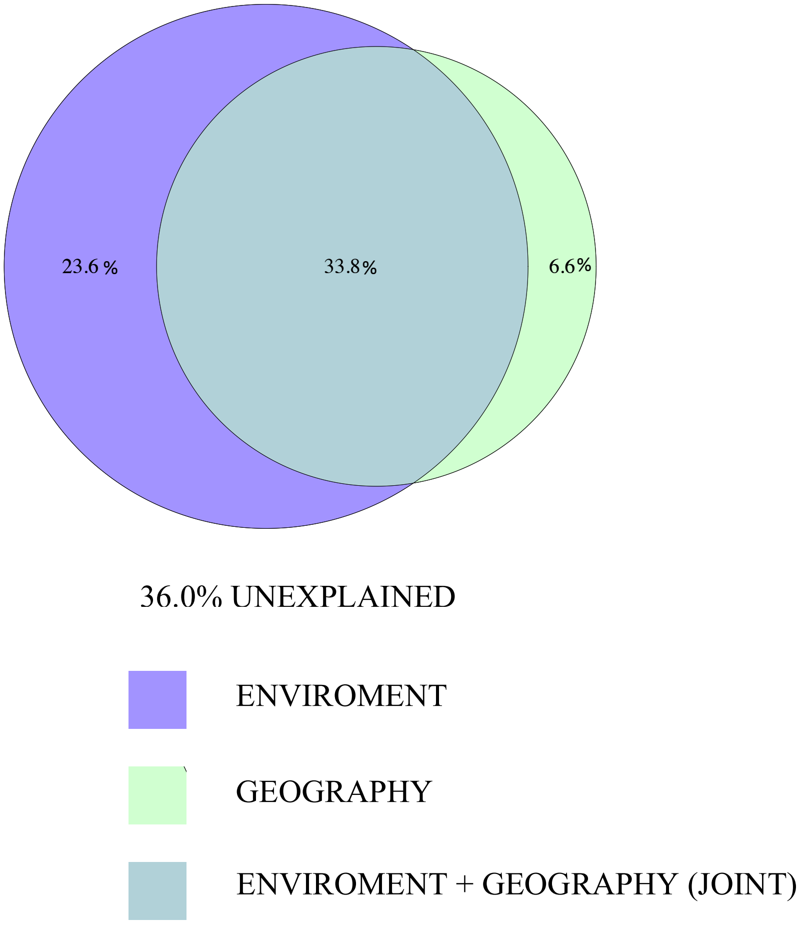

Figure S4. Linear regression plots illustrating the joint effects of PWM on PCOA1 (A) and MDR on PCOA1 (B) at the mean of AP and at 2 standard deviations above and below the AP mean. PWM and MDR were standardized, and used as the exploratory (independent) variables in the regressions. Differences in the regression slopes at different AP values represent interactive effects of the environmental variables on the PCOA1 scores.

A

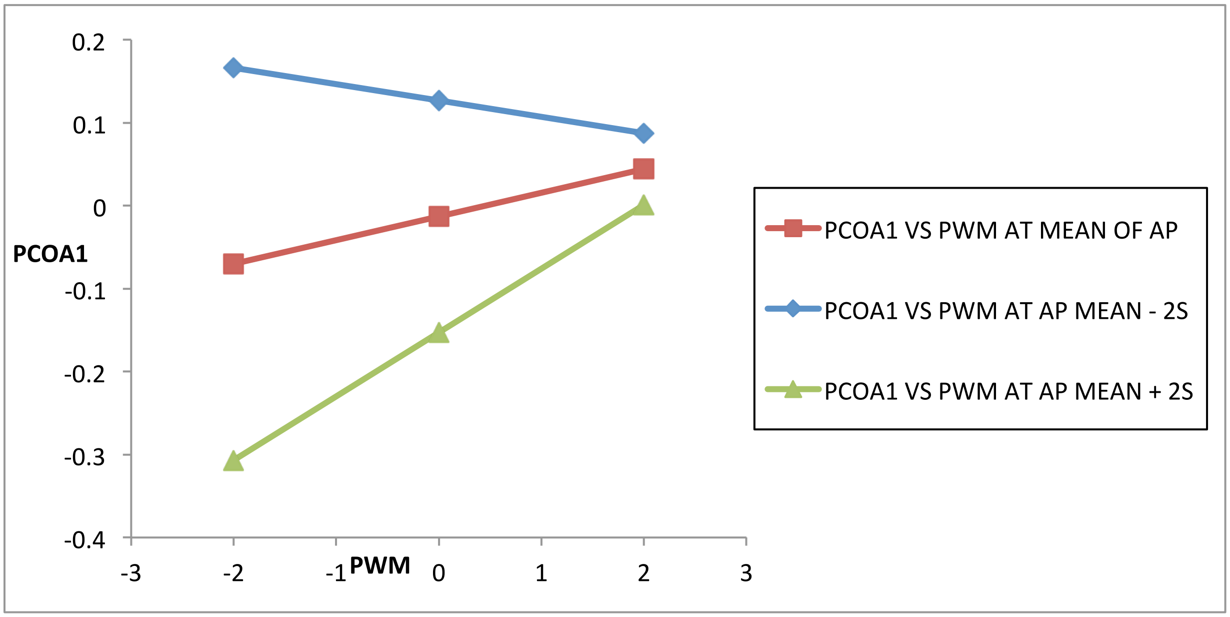

B

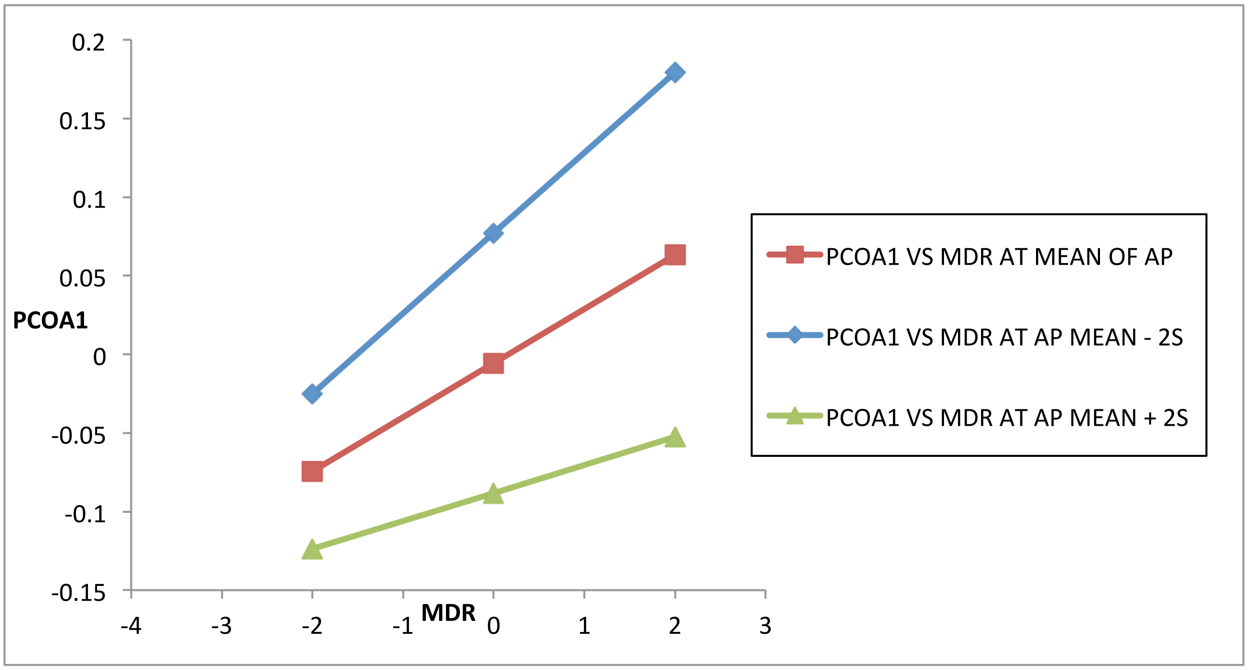

Supplement: Supplementary file 1 — Table S1. Correlations among the geographical and environmental variables for soybean the soybean sample. Table S2. Membership for the 99 soybean accessions in each of the four groups identified by the STRUCTURE procedure. Table S3. Means of the eight (untransformed) geographic and environmental variables for each of the four genetic groups. Figure S1. Statistics from the STRUCTURE analysis (Evanno et al. 2005) of the wild soybean genetic data suggest that the most plausible number of genetic groups (K value) is 4. Figure S2. Neighbor‐joining tree generated from the 99 wild soybean ecotypes. Figure S3. A venn diagram showing the proportion of genomic variance explained by environmental variables (light blue), geography (light green), and the joint effects of environment and geography (blue‐green). Figure S4. Linear regression plots illustrating the joint effects of PWM on PCOA1 (A) and MDR on PCOA1 (B) at the mean of AP and at 2 standard deviations above and below the AP mean. [file ECE3-6-6332-s001.pdf]
